# A preoperative nomogram predicting risk of lymph node metastasis for early-stage cervical cancer

**DOI:** 10.1186/s12905-023-02726-0

**Published:** 2023-11-03

**Authors:** Yuan-Run Deng, Xiao-Jing Chen, Cai-Qiu Xu, Qiao-Zhi Wu, Wan Zhang, Sui-Qun Guo, Li-Xian Li

**Affiliations:** 1 Department of Obstetrics and Gynecology, The Third Affiliated Hospital, Southern Medical University, Tianhe District, 183 Zhongshan Avenue West, Guangzhou, 510630 P. R. China; 2 Department of Obstetrics and Gynecology, The First Affiliated Hospital of Guangzhou Medical University, Guangzhou, 510120 China; 3 Department of Radiation Oncology, Affiliated Dongguan People’s Hospital, Southern Medical University, Dongguan, 523059 China; 4 Department of Medical Matters, Puning People’s Hospital, 30 Liusha Dadao, Puning, 515300 P. R. China

**Keywords:** Cervical cancer, Lymph node metastasis, Lymphadenectomy, Nomogram, SEER

## Abstract

**Objective:**

This study aimed to develop a preoperative nomogram based on clinical and pathological characteristics to provide a more individualized and accurate estimation of lymph node metastasis (LNM) in patients with early-stage cervical cancer.

**Methods:**

A total of 7,349 early-stage cervical cancer patients with pathologically confirmed between 1988 and 2015 were obtained from the Surveillance, Epidemiology, and End Results (SEER) database. All the patients were divided into training (*n* = 5,500) and validation (*n* = 1,849) cohorts randomly. A cohort of 455 patients from multicenter was used for the external validation. We established a multivariate logistic regression model based on preoperative clinicopathological data, from which a nomogram was developed and validated. A predicted probability of LNM < 5% was defined as low risk.

**Results:**

From multivariate logistic regression analysis, age at diagnosis, histologic subtype, tumor grade, tumor size and FIGO stage were identified as preoperative independent risk factors of LNM. The nomogram incorporating these factors demonstrated good discrimination and calibration (concordance index = 0.723; 95% confidence interval (CI), 0.707–0.738). In the validation cohort, the discrimination accuracy was 0.745 (95% CI, 0.720–0.770) and 0.747 (95% CI, 0.690–0.804), respectively. The nomogram was well calibrated with a high concordance probability. We also established an R-enabled Internet browser for LNM risk assessment, which tool may be convenient for physicians.

**Conclusions:**

We developed an effective preoperative nomogram based on clinical and pathological characteristics to predict LNM for early-stage cervical cancer. This model could improve clinical trial design and help physicians to decide whether to perform lymphadenectomy or not.

## Background

Cervical cancer was the second leading cause of cancer death among women worldwide [1]. The vast majority of women with early-stage cervical cancer are associated with a good prognosis; however, a subgroup of women with lymph node metastasis (LNM) are at high risk of relapse and death [2]. LNM is the principal reason for very poor survival of cervical cancer patients [3]. More advances in lymph node (LN) status evaluation are needed pre-operatively.

The standard management of patients with early-stage cervical cancer (FIGO stage I—II) is concurrent chemoradiotherapy or radical hysterectomy (RH) with pelvic and/or para-aortic lymphadenectomy [4]. Adjuvant chemoradiation is often recommended if LNs are found to be positive post surgery. LN involvement is a significant prognostic factor in cervical cancer, which was included in the 2009 FIGO staging system [5]. Thus, information on the status of LN is necessary to determine treatment strategy [6]. Lymphadenectomy has been used to evaluate LN status in cervical cancer. Indications for nodal dissection had been clearly established in all current guidelines at time of hysterectomy being stage 1A1 with lymphovascular space invasion and stage IA2 disease if a primary surgical approach with a radical hysterectomy is chosen for early-stage disease. However, the therapeutic impact of lymphadenectomy is controversial [7]. Only 15–25% of patients with early-stage cervical cancer develop LNM; a large proportion of patients undergo lymphadenectomy unnecessarily and suffer from surgery-related complications [8]. Therefore, it would be useful to identify patients with a low likelihood of LNM preoperatively. Furthermore, the ability to identify low-risk patients may be useful in medical decision management. In other words, patients who are defined as low risk by our nomogram may be considered for exclusion from lymphadenectomy. Considerable effort has been made to reduce the adverse events associated with lymphadenectomy. Recent studies have shown that sentinel LN (SLN) biopsy could be used as a nodal staging method for cervical cancer patients [9]. However, this technique has not been fully validated and requires surgery under general anaesthesia [10]. Therefore, it is of great importance to identify the risk of LNM non-invasively and preoperatively. An evidence-based algorithm for surgical treatment decisions may be helpful, especially when the health status of the patient is intolerable to surgery [11]. Clinical and pathological variables (eg, FIGO stage, histological type and grade) have been reported to be associated with the risk of LNM [12]. However, individually, none of these characteristics can be used to determine the treatment strategy. Prognostic tools, such as nomograms, that combine variables using statistical models to obtain the most reliable and accurate predictions have been adopted by several oncologic disciplines [13]. The aim of this study was to determine the clinical and pathological risk factors for LNM in early-stage cervical cancer patients and to develop a nomogram to predict LNM preoperatively, which may help physicians to make surgical treatment decisions.

## Materials and methods

### Study population

**Fig. 1:**
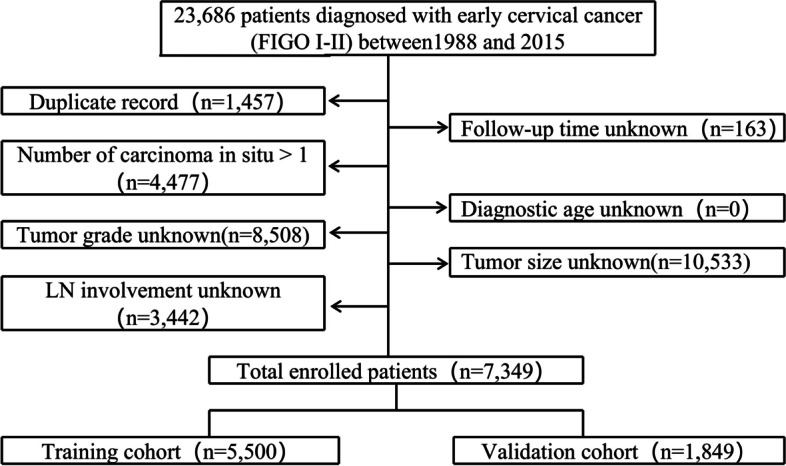
Flow chart. Illustration of patient inclusion

The prediction model was developed using data from the Surveillance, Epidemiology, and End Results (SEER) database, which were publicly available. We identified early-stage cervical cancer patients with pathologically confirmed between 1988 and 2015 (https://seer.cancer.gov/data/). The training cohort of 5,500 patients and the internal validation cohort of 1,849 patients were extracted from the SEER database. Case listings were generated using codes specific for both clinical (i.e., age at diagnosis) and tumor characteristics (i.e., histologic subtype, tumor grade, tumor size, FIGO stage and LNM). The external validation cohort of 455 patients were from multicenter, whose preoperative biopsy were interpreted blindly without knowledge of LN involvement by two experienced pathologists in the respective hospital pathology department. And the 455 patients from multicenter were finally reviewed by a senior pathologist in the Third Affiliated Hospital, Southern Medical University. Tumor size and LN involvement can be determined preoperatively by clinical pelvic examination and imaging, such as CT and MRI. The FIGO classification for staging cervical cancer was based on clinical pelvic examination, combined with imaging and pathological data. The patient selection flow chart was shown in Fig. 1. We included patients diagnosed with early-stage cervical cancer (FIGO stage I—II) between 1988 and 2015 based on the SEER program. Patients with duplicate record or unknown information were excluded (some patients may miss several variables). The exclusion criteria were as follows: (I) patients with duplicate record (*N* = 1,457); (II) number of carcinoma in situ > 1 (*N* = 4477); (III) tumor grade unknown (*N* = 8,508); (IV) LN involvement unknown (*N* = 3,442); (V) follow-up time unknown (*N* = 163); (VI) diagnostic unknown (*N* = 0); (VII) tumor size unknown (*N* = 10,533). Ultimately, 7,349 patients satisfied the eligibility criteria were involved in this analysis. These patients were randomly divided into a training cohort (*N* = 5,500) and an internal validation cohort (*N* = 1,849) at a ratio of 3:1. Additionally, a retrospective Chinese patient cohort consisting of 455 early-stage cervical cancer (FIGO stage I—II) patients from multicenter were included in the external validation set. As the patient information involved in this retrospective analysis came from the routine medical records, our study met the requirement of informed consent exemption. Using SEER data does not require additional informed consent as patient privacy information is protected by the SEER cancer registries.

### Development of the nomogram

The nomogram was established as previously described [11]. Before developing the nomogram, low risk was defined as a predicted probability of developing LNM of < 5% [14, 15]. To develop a well-calibrated and exportable nomogram predicting the risk of LNM, we established a logistic regression model (LRM) using a training cohort of 5,500 patients, which was extracted from the SEER database, and we validated the model with an internal validation cohort of 1,849 patients and an independent external validation cohort of 455 patients. Multivariate logistic regression analyses were used to test the association between the LNM risk and clinicopathological characteristics. The following variables were included in the analysis: age at diagnosis (< 45 years and >  = 45 years); histological subtype (squamous, adenocarcinoma and others); tumor grade (I, II, III-IV); tumor size (< 2 cm, 2 ~ 4 cm, 4 ~ 6 cm, > 6 cm) and FIGO stage (I, II).

The predictive accuracy of the model was assessed in terms of its discrimination and calibration. Discrimination is the ability to distinguish between patients with positive LNM and those with negative LNM, and it is measured using the receiver operating characteristic curve and summarized by the area under the curve (AUC). An AUC of 1.0 indicates perfect concordance, whereas an AUC of 0.5 indicates no relationship. Calibration was studied using graphical representations of the relationship between the observed outcome frequencies and the predicted probabilities, and the Hosmer–Lemeshow test was employed to assess calibration.

### Validation

An internal validation of the accuracy estimates was performed with 1,849 patients to obtain relatively unbiased estimates. For external validation, the model was applied on a sample of 455 patients referred to as the validation set, which was developed from database from 3 institutions: Third Affiliated Hospital, Southern Medical University (155 patients), Dongguan People's Hospital (150 patients) and Puning People’s Hospital (150 patients).

### Other statistical tests

The categorical and numerical variables were analyzed using the χ2 test. A *P* < 0.05 was considered significant. All analyses were performed using R version 3.6.1 (http://cran.r-project.org/mirrors.html).

## Results

### Patient population

The overall data from the 5,500 patients in the training set, the 1,849 patients in the internal validation set, and the 455 patients in the external validation set were analyzed. Patient characteristics are summarized in Table 1. The LNM rates for the training, internal and external validation sets were 18.65% (1,026 of 5,500), 19.09% (353 of 1,849), and 14.07% (64 of 455) respectively.

**Table 1:** Characteristics of the study population

Variables	Training set (*N* = 5500)	Internal Validation set (*N* = 1849)	External Validation set (*N* = 455)
Age at diagnosis			
< 45 years	2870 (52.18%)	984 (52.33%)	171 (37.58%)
> = 45 years	2670 (47.82%)	865 (46.78%)	284 (62.42%)
Histologic subtype			
Squamous	3483 (63.33%)	1214 (65.66%)	414 (90.99%)
Adenocarcinoma	1553 (28.24%)	495 (26.77%)	37 (8.13%)
Others	464 (8.44%)	140 ( 7.57%)	4 (0.88%)
Tumor grade			
I	824 (14.98%)	294 (15.90%)	44 (9.67%)
II	2449 (44.53%)	800 (43.27%)	158 (34.73%)
III or IV	2227 (40.49%)	755 (40.83%)	253 (55.60%)
Tumor size			
< 2 cm	1956 (35.56%)	668 (36.13%)	148 (32.53%)
2 ~ 4(< 4)cm	1572 (28.58%)	528 (28.56%)	205 (45.05%)
4 ~ 6(< 6)cm	1157 (21.04%)	373 (20.17%)	85 (18.86%)
> 6 cm	815 (14.82%)	280 (15.14%)	17 (3.74%)
FIGO			
I	3930 (71.45%)	1297 (70.15%)	271 (59.56%)
II	1570 (28.55%)	552 (29.85%)	184 (40.44%)
LNM			
No	4470 (81.27%)	1496 (80.91%)	391 (85.93%)
Yes	1026 (18.65%)	353 (19.09%)	64 (14.07%)

*FIGO* International Federation of Gynecology and Obstetrics, *LNM* lymph node metastasis

### A nomogram for the prediction of LNM

**Fig. 2:**
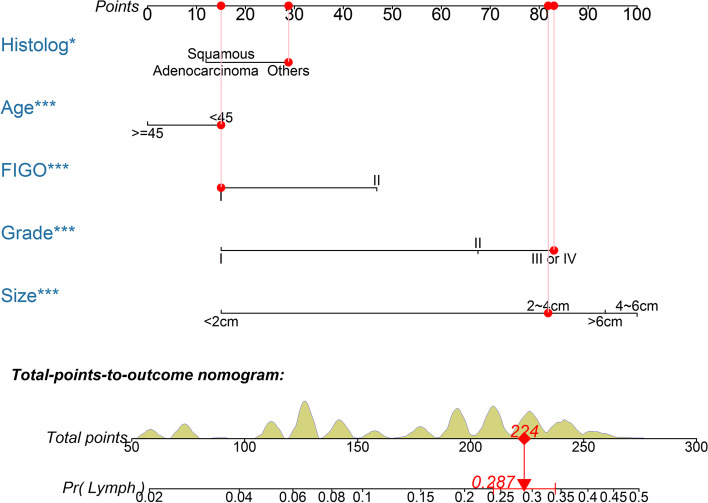
Nomogram predicting LNM in patients with early-stage cervical cancer. The probability of metastatic lymph node involvement is calculated by drawing a line to the point on the axis for each of the following variables: Age at diagnosis, histologic subtype, tumor grade, tumor size, and FIGO stage. The points for each variable are summed and located on the total points line. Next, a vertical line is projected from the total points line to the predicted probability bottom scale to obtain the individual probability of metastatic lymph node involvement. A total score of 110 was assigned a value of 0.05 and was defined as low risk for LNM. Grade I = Well differentiated; Grade II = Moderately differentiated; Grade III = Poorly differentiated; Grade IV = Undifferentiated

Table 2 summarizes the multivariate logistic regression analyses. The metastatic LN risk was independently associated with age at diagnosis, histologic subtype, tumor grade, tumor size and FIGO stage. The multivariate logistic regression analysis showed that patients with older age (> = 45 ys) had a 0.76-fold risk for LNM (95% CI 0.66–0.88; *P* < 0.001). In addition, patients with non-squamous and non-adenocarcinoma histologic subtype had a 1.28-fold increased risk for LNM (95% CI 1.01–1.62; *P* = 0.045), patients with grade II and III or IV had a 2.57-fold (95% CI 1.83–3.60; *P* < 0.001) and 3.39-fold (95% CI 2.41–4.77; *P* < 0.001) increased risk for LNM respectively, patients with tumor size 2 ~ 4 cm, 4 ~ 6 cm and > 6 cm had a 3.32-fold (95% CI 2.63–4.18; *P* < 0.001), 4.60-fold (95% CI 3.61–5.87; *P* < 0.001) and 4.10-fold (95% CI 3.14–5.34; *P* < 0.001) increased risk for LNM respectively, and patients with higher FIGO stage (FIGO II) had a 1.77-fold increased risk for LNM (95% CI 1.50–2.08; *P* < 0.001) (Table 2). The nomogram constructed from the final multivariate model is presented in Fig. 2. For a given patient, points are assigned to each of the predictor variables in the nomogram and a total score is derived from the sum of present variables. The total point score corresponds to a predicted probability of LNM.

**Table 2:** Predictors of metastatic lymph nodes in multivariable analysis

Variables	OR(95%CI)	*P* value	Wald *P* value
(Intercept)	0.03 (0.02, 0.04)	< 0.001	< 0.001
Age at diagnosis			0.001
< 45 ys	Referent		
> = 45 ys	0.76 (0.66, 0.88)	< 0.001	
Histologic subtype			0.045
Squamous	Referent		
Adenocarcinoma	0.95 (0.79, 1.13)	0.043	
Others	1.28 (1.01, 1.62)	0.039	
Tumor grade			< 0.001
I	Referent		
II	2.57 (1.83, 3.60)	< 0.001	
III or IV	3.39 (2.41, 4.77)	< 0.001	
Tumor size			< 0.001
< 2 cm	Referent		
2 ~ 4(< 4)cm	3.32 (2.63, 4.18)	< 0.001	
4 ~ 6(< 6)cm	4.60 (3.61, 5.87)	< 0.001	
> 6 cm	4.10 (3.14, 5.34)	< 0.001	
FIGO			< 0.001
I	Referent		
II	1.77 (1.50, 2.08)	< 0.001	

*FIGO* International Federation of Gynecology and Obstetrics, *CI* confidence interval, *OR* odds ratio

### Validation of the nomogram

#### Discrimination

**Fig. 3:**
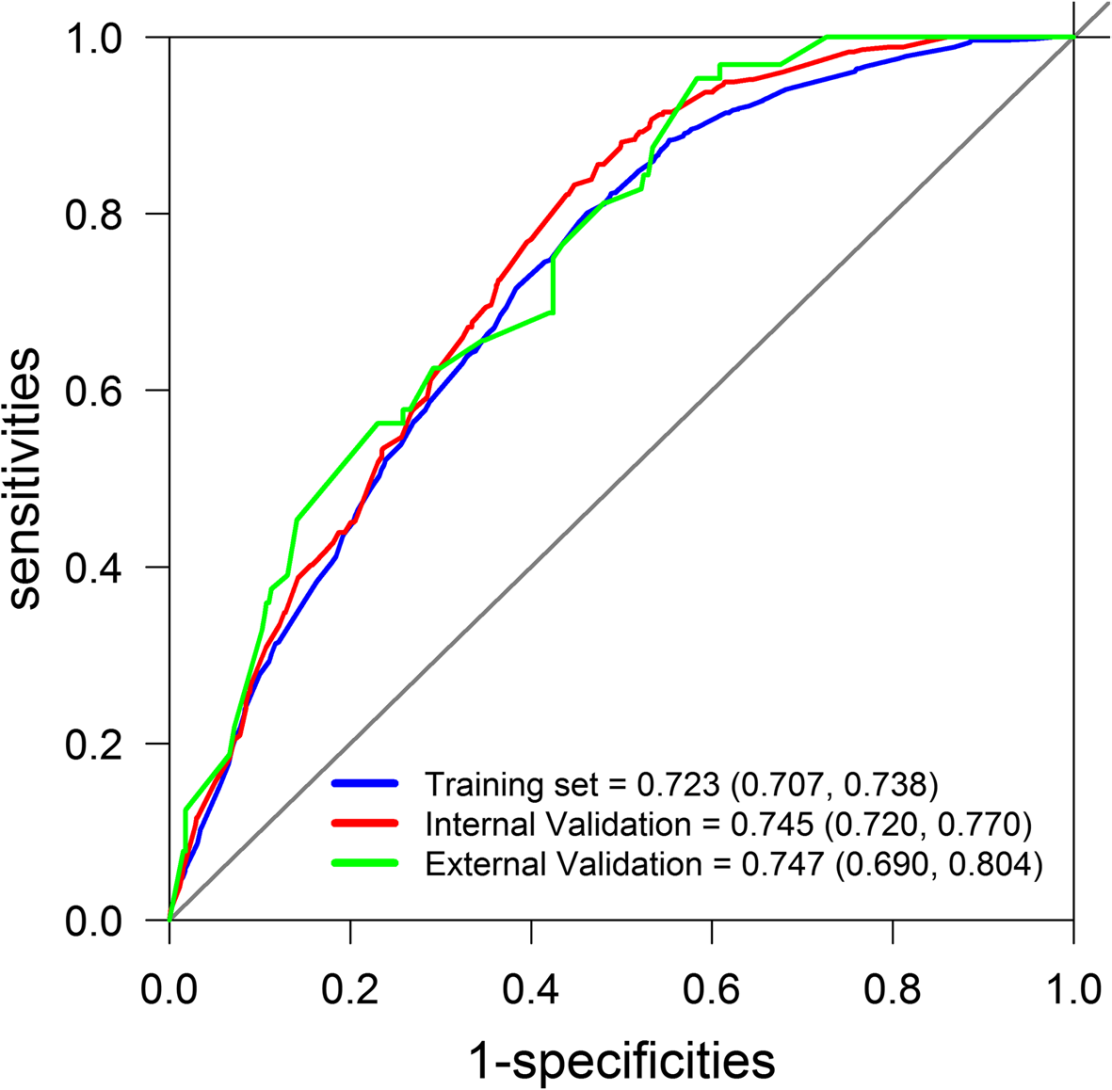
Receiver operating characteristic curves of the model. In the training set, the AUC was 0.723 (95% CI, 0.707–0.738) (blue line); in the internal validation set, the AUC was 0.745 (95% CI, 0.720–0.770) (red line); in the external validation set, the AUC was 0.747(95% CI, 0.690–0.804) (green line). AUC, area under the curve; CI, confidence interval

First, the nomogram was validated using the correction technique. The corrected concordance index for the model was 0.723 (95% CI, 0.707–0.738) (Fig. 3, blue line). In the internal validation set, the discrimination accuracy of the model was 0.745 (95% CI, 0.720–0.770) (Fig. 3, red line). In the external validation set, the discrimination accuracy of the model was 0.747 (95% CI, 0.690–0.804) (Fig. 3, green line).

#### Calibration

**Fig. 4:**
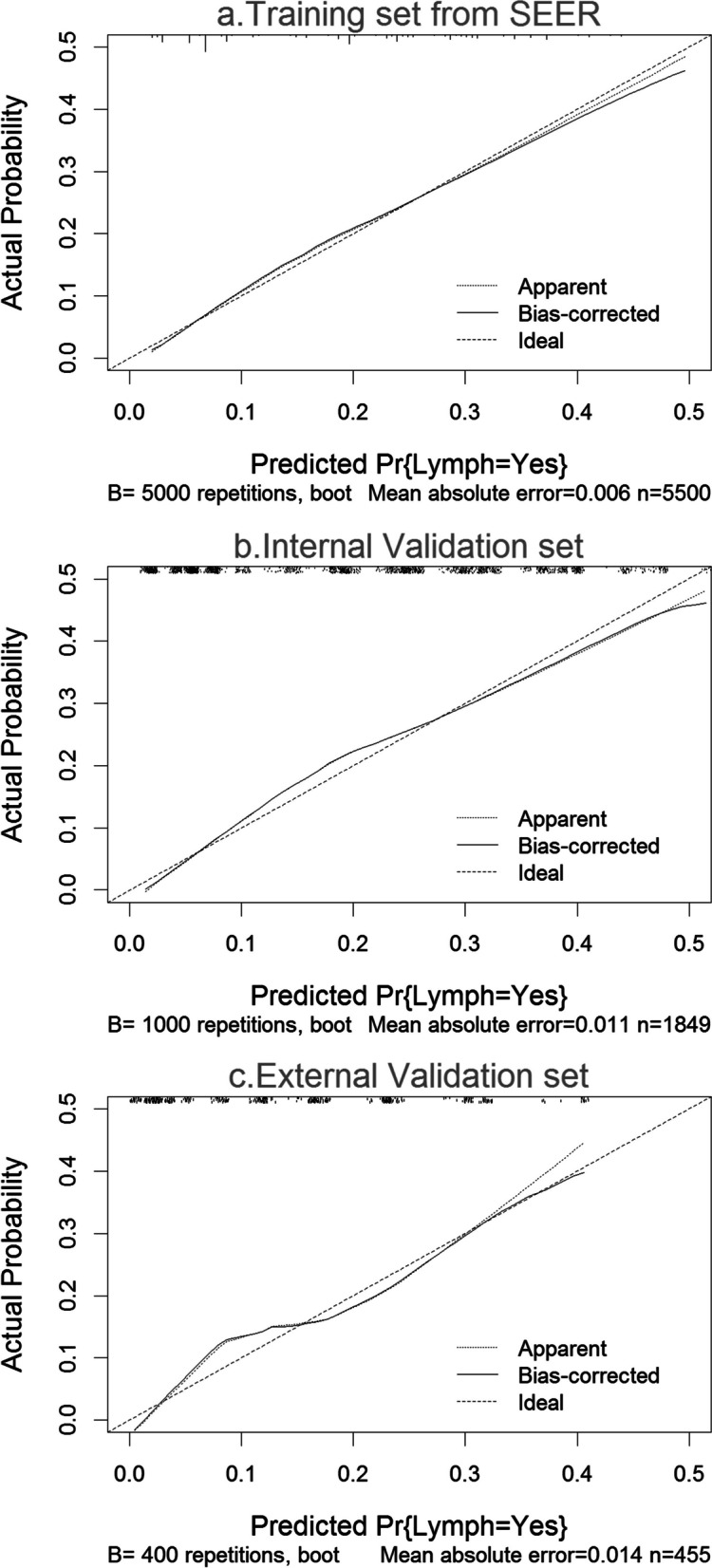
Calibration plot of the nomogram to predict metastatic lymph node involvement. **A** Calibration plots of the nomogram for the training cohort. **B** Calibration plots of the nomogram for the internal validation cohort. **C** Calibration plots of the nomogram for the external validation cohort. Dashed line = ideal reference value where predicted probabilities match actual probabilities of LNM; solid line indicates performance of the current nomogram; filled dots indicate calculations from a subcohort of the present database

Figure 4 show the calibration plots of the nomogram for the training and validation cohorts, respectively. The dashed line represents the performance of the ideal nomogram and the solid line represents the performance of the current nomogram. The filled points were derived from a subgroup of the current database. When comparing the LNM probability predicted by the nomogram against the actual probabilities, the calibration curve is located near the dashed line. There was no difference between the predicted probability and the observed rate of LNM (*P* > 0.05). The Hosmer–Lemeshow test yielded a *P*-value of 0.363 for the model-development cohort, showing that the nomogram was well fitted. For the validation cohort, the nomogram also fitted the data well (*P* = 0.219 for internal validation, *P* = 0.427 for external validation, Hosmer–Lemeshow test).

### Identification of patients at low risk of LNM

The low-risk group was predefined as having a predicted probability of < 5%. The nomogram classified 389 out of 1849 patients (21.03%) in the internal validation cohort as low risk. In that group, the predicted probability of LNM was 3.52% and the actual metastasis rate was 3.08% (12 out of 389). In the external validation cohort, 82 out of 455 patients (18.02%) were classified as low risk. In that group, the predicted probability of LNM was 4.20% and the actual metastasis rate was 3.65% (3 out of 82).

### A R-enabled Internet browser for LNM risk assessment

**Fig. 5:**
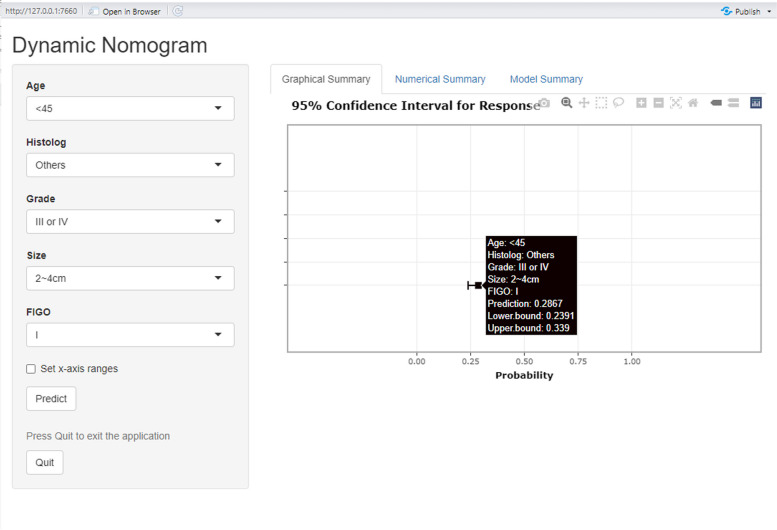
A R-enabled Internet browser for LNM risk assessment. Example of a screen from a computer program

We developed an algorithm that uses the prediction models described above to estimate the risk of LNM in individual cervical cancer patient. The predictors were programmed in R project. Running the applets requires an Internet browser that supports R. This R-enabled Internet browser could provide exact estimate of LNM risk in the output, which may help physicians to make decisions regarding lymphadenectomy. Example of a screen from the computer program is shown in Fig. 5. Table 3 shows a brief description of the three patients who underwent LNM risk assessment using our nomogram.

**Table 3:** A brief description of the three patients of LNM risk assessment with our nomogram

Characteristics	Patient 1		Patient 2		Patient 3	
	**Information**	**Score**	**Information**	**Score**	**Information**	**Score**
**Age at diagnosis**	> 45	2	< 45	15	< 45	15
**Histologic subtype**	Squamous	10	Other	30	Squamous	10
**Tumor grade**	I	15	I	15	III	83
**Tumor size**	< 2	15	< 2	15	> 6	95
**FIGO**	I	15	I	15	II	50
**Total**		57		90		253
**Predicted LNM**	2.0%		3.6%		40%	
**Actual LNM**	NO		NO		YES	

*FIGO* International Federation of Gynecology and Obstetrics, *LNM* lymph node metastasis

## Discussion

Our study suggests that the risk assessment of LNM could be made before surgery using clinical and pathological characteristics, including age at diagnosis, histologic subtype, tumor grade, tumor size and FIGO stage. Incorporating these five variables, a preoperative nomogram predicting risk of LNM was developed, in which patients with predicted probability of LNM < 5% were defined as a low-risk group. In the validation cohorts, the LNM rate defined by our nomogram was close to the actual rate. In this regard, lymphadenectomy could be decided according to this nomogram.

Based on accurate assessment of LN status, the extent of lymphadenectomy can be individualized. Previous studies have found that advanced FIGO stage, large tumor size, involvement of the parametrium, and lymphovascular invasion is an independent risk factor for LNM in cervical cancer [16, 17]. But these studies did not combine the independent risk factors together for analysis. And the risk of LNM may be more accurately predicted by incorporating these modalities into the nomogram. Individualised prediction based on the nomogram could provide information for physicians and patients' decision-making. In addition, the most important benefit of nomogram is that the risk of LNM can be assessed by preoperatively with non-invasive procedures. Kim et al*.* developed a nomogram to predict LNM in cervical cancer patients before hysterectomy based on the age, tumor size assessed by MRI, and LNM assessed by PET/CT [15]. But their model was developed using a small sample (304 cases in model-development cohort, 189 cases in validation cohort), and there is no evidence of its generalizability. Although PET/CT and MRI could be useful in detecting LN metastases, the accuracy decreases for nodal size < 5 mm and micrometastasis [18, 19]. In addition, PET/MRI-diffusion weighted imaging may be a valuable imaging technique for nodal staging in cervical cancer patients, but its potential clinical applications still require further research [20]. In this study, we propose a preoperative nomogram based on clinical and routinely definitive pathological characteristics to estimate the risk of metastatic LN involvement. The good predictive value and the simplicity of the tool are important factors to ensure its clinical application and popularization. We tested its general applicability in a multicenter independent population. The predictive accuracy and validity characteristics of this tool were related to a high probability of consistency.

Although lymphadenectomy is the standard criterion for evaluating the LN status of cervical cancer, the therapeutic value of this surgery is controversial, and there are no guidelines regarding the type and extent of LN dissection [7]. The decision to perform a systematic lymphadenectomy depends on the tumor characteristics of the primary sites and LNs [21]. Theoretically, patients with negative LNM will not benefit from lymphadenectomy [22]. Lymphadenectomy may lead to complications such as lymphocysts, lymphoedema, vessel damage, nerve injuries, and infection [23]. Since the LNM rate in early-stage cervical cancer is 15–25%, about 80% of the patients who undergo lymphadenectomy may have little benefit [24]. Given the adverse events of lymphadenectomy are likely to occur more frequently, we believe that the decision to perform lymphadenectomy should be based on an accurate and individualized risk assessment of LNM. Recent data suggest that sentinel lymph node (SLN) biopsy is an important index for evaluating pelvic nodal status in cervical cancer, with good detection rates and high sensitivity [9]. However, SLN biopsy is not routinely performed due to the lack of consistent data on intraoperative pathological evaluation, the role of micrometastasis in LNs, and surgical criteria [10, 25]. In addition, the role of SLN biopsy and the prognostic value of metastatic LN resection should be evaluated in the risk group rather than in the entire population [26]. Our study suggests that patients who are defined as low risk by our nomogram should be excluded from such trials. With respect to diagnostic performance, our nomogram prediction was comparable to data from a recently published prospective, multicentre SLN biopsy study. The SENTICOL study showed that given a LNM prevalence of 17.9%, the detection sensitivity was 82.0% [27], which is similar to the performance observed in our study. We believe that we could identify the node-negative patients more accurately by combined use of SLN biopsy and the nomogram.

Personalized risk assessment is particularly important in the presence of substantial comorbidities because it informs the discussion of the benefit of the procedure based on the patient's health status and LNM risk [28]. Before evaluation of this nomogram with preoperative tumor characteristics, it can be used only when hysterectomy was performed and lymphadenectomy was omitted [29]. Surgeons may be reluctant to perform a lymphadenectomy except in frail patients, and there is at least one situation that may prevent surgeons from performing lymphadenectomy: when the tumor grade and/or stage have been underestimated. In this case, our nomogram can be used to assess the risk of LNM and to decide whether to proceed with secondary lymphadenectomy or not.

These results do not indicate that routine lymphadenectomy is beneficial for non-low-risk patients. Although the actual LNM rate was 30.6% in the non-low-risk group, the therapeutic value of lymphadenectomy in this group must be evaluated in clinical trials, and the general condition of the patient should always be considered. In addition, the role of SLN biopsy and the prognostic value of metastatic nodal resection should be assessed in this risk group and not in the entire population. In our study, the actual LNM rate of the low-risk group defined by our nomogram in the validation cohorts was remarkably low. Hence, patients defined as low risk by our nomogram should be excluded from such trials.

Our research has several limitations that need to be acknowledged. First, inherent bias is inevitable in retrospective studies. Second, the SEER program lacks data concerning several known factors associated with LNM, such as lymphovascular invasion (LVSI), human papillomavirus (HPV), and stromal invasion, which may cause bias to analysis. If these variables can be accurately estimated and included in this model, the performance index can be improved. In addition, due to the lack of detailed record in the SEER database, the extents of lymphadenectomy (pelvic or para-aortic) were not clearly defined. Third, unlike databases created by random sampling of the target populations, SEER is not a random sample of the entire population of cancer patients in the United States [30]. Fourth, we did not use double-programming in the current data analysis, which may increase the reliability of the conclusion although not necessary. Fifth, our study has general limitations of logistic regression, such as inability to solve multicollinearity problems, limited ability to adapt to data and scenarios. However, the advantage of this study is that it analyzed a large number of patient data from the SEER project, which was specifically designed to provide population-based data. In the follow-up study, we will collect the updated retrospective data and expand the sample size for external verification. And we will also conduct a prospective validation study in large, heterogeneous populations to evaluate the predictive accuracy of the model in future research.

## Conclusions

In conclusion, we constructed and validated an effective and convenient preoperative model for predicting LNM in patients with early-stage cervical cancer. This nomogram incorporated important prognostic factors to provide a more accurate prediction of individual patient risk. The high consistency probability of this nomogram was verified in an independent, external, and multicenter dataset. This new tool may be useful for clinicians and patients in deciding whether to proceed with lymphadenectomy, as well as for designing clinical trials.

## Data Availability

Data from the SEER program is available for the public. The data supporting the conclusions of this article are available in the Surveillance Epidemiology, and End Results (SEER) database (https://seer.cancer.gov/).

## Abbreviations

LNM: Lymph node metastasis
SEER: Surveillance, Epidemiology, and End Results
CI: Confidence interval
RH: Radical hysterectomy
SLN: Sentinel lymph node
LRM: Logistic regression model
AUC: Area under the curve
NPV: Negative predictive value
LVSI: Lymphovascular space invasion
